# Wilkie’s syndrome as the cause of intestinal obstruction in an 18-year-old female adolescent: A case report

**DOI:** 10.1016/j.radcr.2026.04.045

**Published:** 2026-05-17

**Authors:** Wondwosen Mengist Dereje, Muluken Assefa Zemariam, Melese Birara Mequanint, Yonas Admaw Tiruneh, Desalegn Kefale Aegash, Melaku Tessema Kassie, Asratu Getnet Amare, Ashenafi Amsalu Feleke

**Affiliations:** a College of Medicine and Health sciences, Department of Neurology, University of Gondar, Gondar, Ethiopia; b College of Medicine and Health sciences, Department of Surgery, University of Gondar, Gondar, Ethiopia

**Keywords:** Wilkie’s syndrome, Bowel obstruction, Superior mesenteric artery, Strong’s procedure, Case report

## Abstract

Wilkie’s syndrome, also known as superior mesenteric artery (SMA) syndrome, is an extremely rare clinical condition encountered in medical practice. It occurs when the third part of the duodenum is compressed between the SMA anteriorly and the aorta and vertebral column posteriorly, leading to partial or complete bowel obstruction. An 18-year-old female adolescent presented with a 2-week history of vomiting. She also complained of crampy abdominal pain and progressive abdominal distension of the same duration. Her symptoms began after she underwent an appendectomy for acute appendicitis. Upon presentation, she was in severe pain and tachycardic, with a pulse rate of 126 beats per minute. Examination revealed a distended abdomen with isolated epigastric tenderness. Plain abdominal radiography was performed and was suggestive of small bowel obstruction. A computed tomography scan of the abdomen was then done, which showed superior mesenteric artery syndrome (Wilkie’s syndrome). After discussion with the patient and her family laparotomy was performed. Intraoperatively, the third part of the duodenum was found to be compressed between the superior mesenteric artery anteriorly and aorta and vertebral column posteriorly. A modified Strong’s procedure was performed. Postoperatively, she remained stable, and after 5 days in the hospital, she was discharged with dietary advice. At her outpatient follow-up appointments at 2 and 4 weeks post-discharge, she showed clinical improvement, with no signs of malnutrition, and was subsequently discharged from follow-up. Despite being a rare cause of obstruction, Wilkie’s syndrome should be considered as a differential diagnosis in patients with chronic illness or a history of prior surgery.

## Introduction

Vascular compression of the duodenum was first described by Rokitansky in 1842 [1,2]. Superior mesenteric artery (SMA) syndrome, also known as Wilkie’s syndrome, is a rare but recognized cause of small bowel obstruction, although its precise incidence remains unknown. The condition results from a narrowing of the aortomesenteric angle [3]. Despite its rarity, it represents a potentially life-threatening gastrointestinal disorder [4].

## Case report

An 18-year-old female presented with a 2-week history of postprandial vomiting, occurring 1-2 times per day. She also reported non-localized, crampy abdominal pain of the same duration.

Two weeks earlier, she had been diagnosed with acute appendicitis after presenting with a 2-day history of crampy abdominal pain. An appendectomy was performed without complications. On the third postoperative day, she developed crampy abdominal pain and postprandial vomiting. Investigations revealed mild hypokalemia (3.1 mmol/L). She was treated with intravenous potassium chloride, her potassium level was corrected, and the crampy abdominal pain and vomiting subsequently subsided.

She was discharged with advice and a scheduled follow-up appointment at the surgical outpatient clinic.

Two weeks after discharge, just prior to her outpatient visit, she presented to the emergency department with repeated vomiting of ingested material, crampy abdominal pain, and abdominal distension. She reported experiencing intermittent vomiting over the preceding period but had not sought medical attention, hoping the symptoms would resolve spontaneously. She also noted unquantified but significant weight loss.

On presentation, she appeared acutely ill and in severe pain, actively vomiting. Her vital signs were: blood pressure 100/60 mmHg, pulse rate 126 bpm, respiratory rate 19 breaths per minute, temperature 36.9°C, and oxygen saturation 98% on room air.

Anthropometric assessment revealed a BMI of 17.2 kg/m², indicative of malnutrition. Physical examination showed a distended abdomen that moved with respiration and audible bowel sounds of 14 gurgles per minute. She had isolated epigastric tenderness without tenderness in the other abdominal quadrants. The remainder of the systemic examination was unremarkable.

**Fig. 1:**
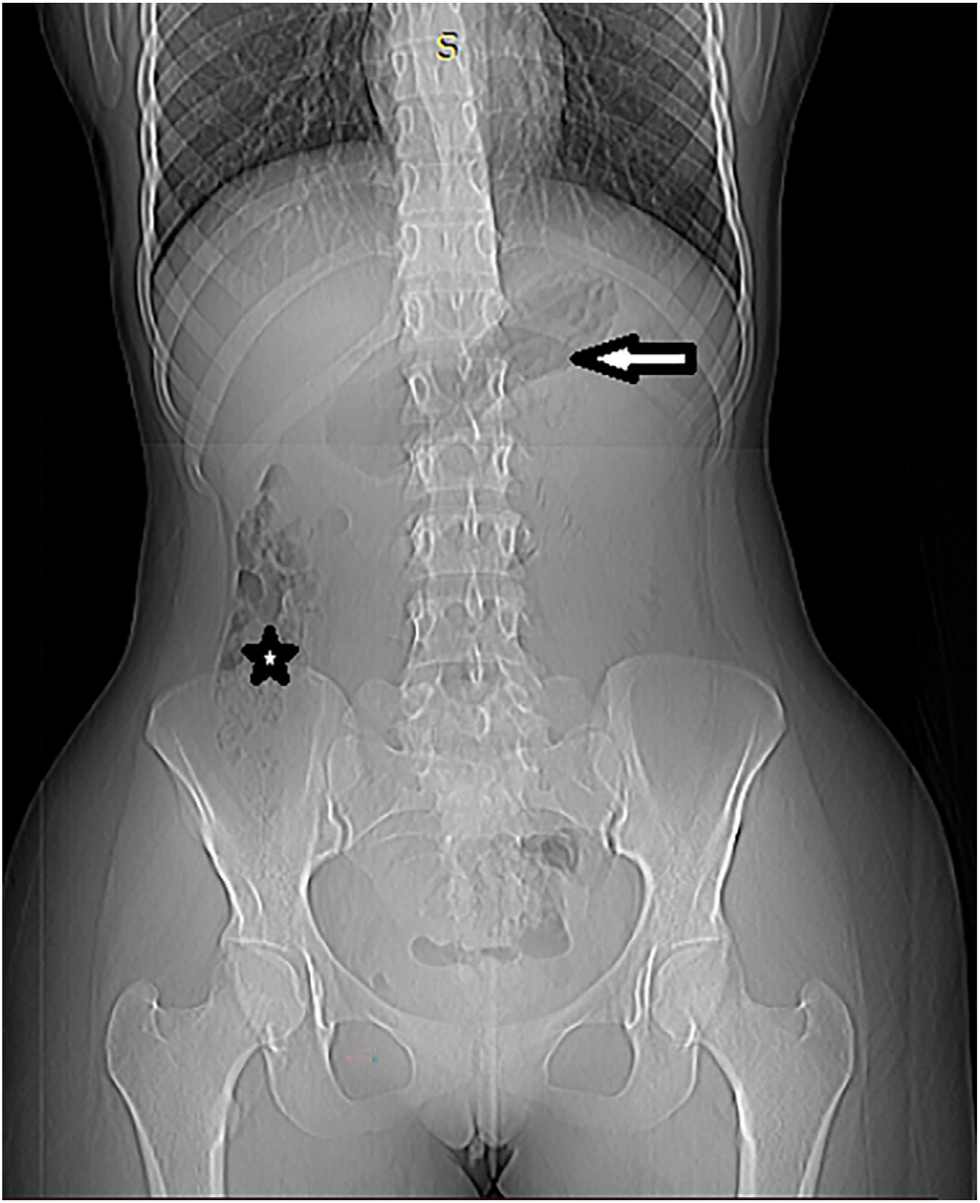
Plain abdominal radiography showing dilated stomach (arrow) and duodenum (star).

A plain abdominal radiograph demonstrated dilated stomach and duodenum (Fig. 1), suggestive of bowel obstruction.

**Fig. 2:**
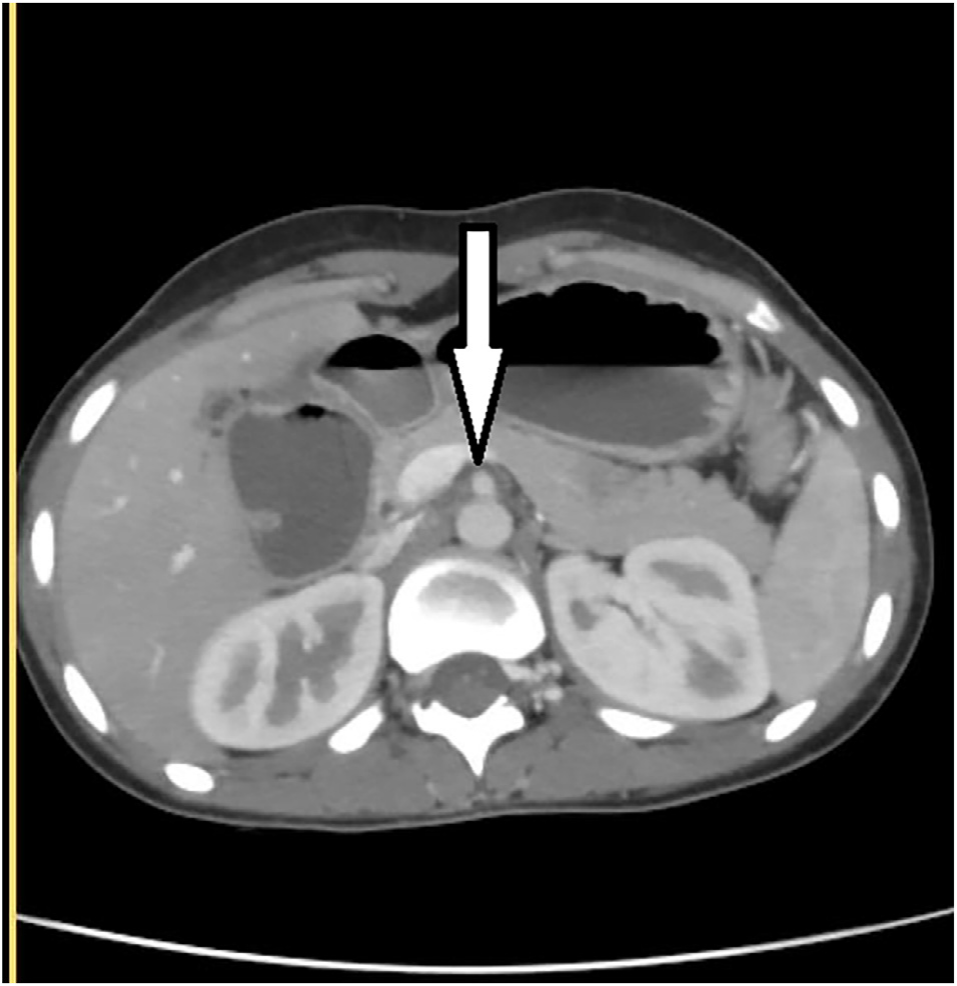
Axial plane; contrast enhanced CT scan of the abdomen (portal venous phase) showing SMA arising from abdominal aorta (arrow).

**Fig. 3:**
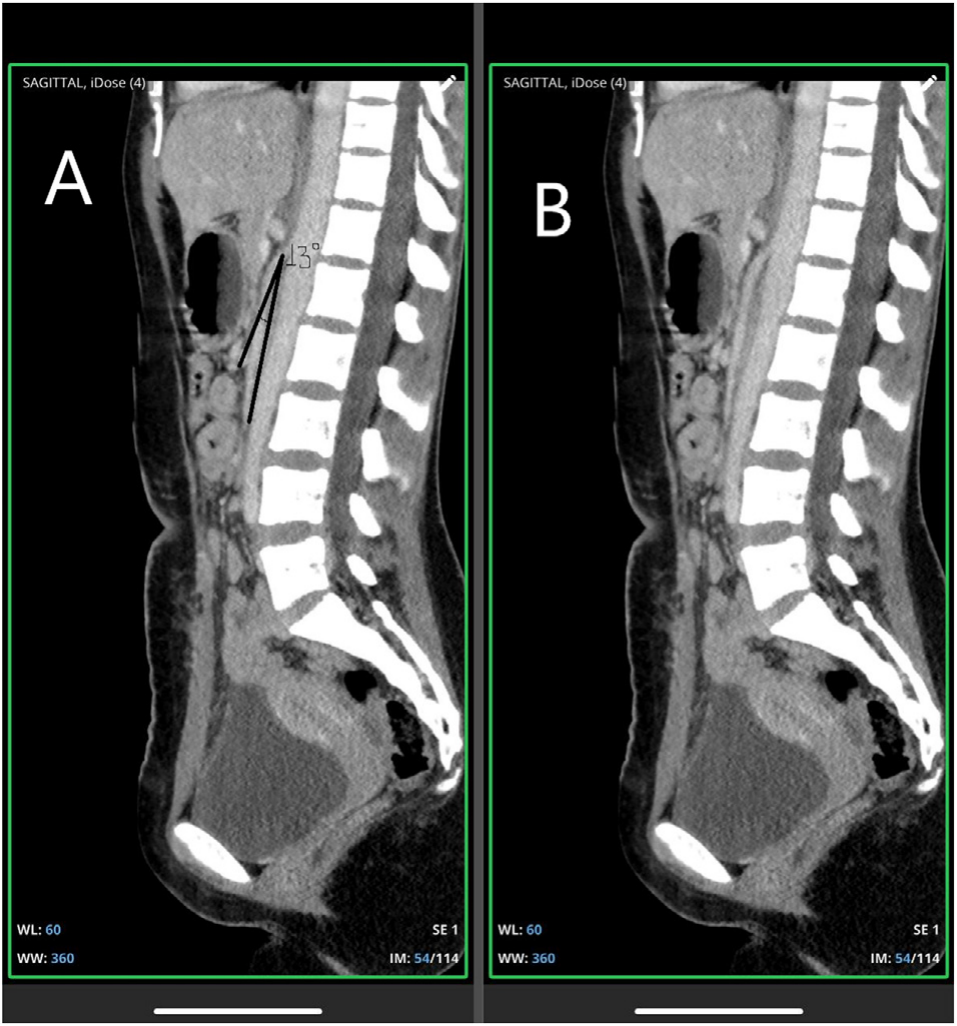
A-B: Saggital plane; CT scan of the abdomen with narrowed aorto-SMA angle (13^0^) (A) and clear version without annotation (B).

A contrast-enhanced CT scan of the abdomen revealed a dilated stomach and proximal duodenum and compression of the third portion of the duodenum at the level of the superior mesenteric artery (SMA) and SMA arising from aorta (Fig. 2), with a narrowed aorto-SMA distance of 4 mm and an angle of 13° (Figs. 3A and B).

The findings were discussed with the patient and her family. Given the prolonged clinical course, surgical intervention was recommended and they consented. After obtaining informed consent, the patient was taken to the operating room for laparotomy.

Intraoperatively, the third portion of the duodenum was compressed between the SMA anteriorly and aorta and vertebral column posteriorly, with dilation of the proximal duodenum and stomach. A modified Strong’s procedure was performed.

Postoperatively, the patient was transferred to the PACU with stable vital signs, where she remained for 12 hours before being moved to the recovery room. Forty-eight hours after surgery, she was transferred to the ward. She remained in the ward for 5 days, tolerated oral feeding well, and had no complaints. She was subsequently discharged with a scheduled follow-up appointment at the surgical outpatient clinic.

## Discussion

SMA syndrome is a rare condition, with reported incidence ranging from 0.0024%-0.34% in the general population [5]. Wilkie's syndrome develops when the third portion of the duodenum becomes compressed between the SMA and the aorta. Normally, the angle between the SMA and the aorta ranges from 25°-60°, but this angle becomes significantly narrowed in affected individuals [6].

Anatomically, the typical aortomesenteric angle measures between 25° and 60°, and the normal aortomesenteric distance ranges from 10 mm-28 mm. In this case, these measurements were reduced to 13° and 6 mm, respectively. The third part of the duodenum lies just posteroinferior to the SMA, and any reduction in retroperitoneal fat can decrease this angle, ultimately leading to Wilkie’s syndrome [7,8].

The aortomesenteric angle may become narrowed due to various factors, including congenital anomalies, significant weight loss, lumbar hyperlordosis, or restorative proctocolectomy with ileal-anal anastomosis [[9], [10], [11]]. SMA syndrome is frequently associated with severe, chronic, and debilitating conditions such as cancer, malabsorption syndromes, AIDS, trauma, burns, and even surgical procedures, particularly those that alter normal anatomical relationships [12]. The condition most commonly affects females between 10 and 40 years of age [13].

Clinical manifestations of Wilkie's syndrome are generally vague and non-specific. The most common symptoms include post-prandial abdominal pain (59%), nausea (40%), vomiting (50%), early satiety (32%), and anorexia (18%). These symptoms typically worsen when the patient lies supine after eating and improve when assuming the left lateral decubitus, prone, or knee-chest position [14].

In addition, patients may present with acute signs of bowel obstruction, as in this case, though more frequently they exhibit chronic symptoms such as recurrent abdominal distention, abdominal pain, postprandial fullness, or early satiety. Pain often intensifies in the supine position; consequently, patients commonly adopt a knee-chest posture after meals to widen the aortomesenteric angle. This temporarily relieves the partial duodenal obstruction and facilitates the passage of gastric contents through the narrowed segment [13].

Because of its insidious onset, diagnosing SMA syndrome can be particularly challenging. A correct diagnosis relies on a thorough assessment of the patient’s medical history and clinical presentation, supported by appropriate imaging studies. Barium studies may reveal dilation of the duodenum and even the stomach, along with delayed gastroduodenojejunal transit. Endoscopy can demonstrate extrinsic compression and narrowing at the level of the third portion of the duodenum. Contrast-enhanced CT or magnetic resonance angiography provides clear visualization of the vascular compression and allows precise measurement of the aortomesenteric angle and distance—information that is crucial both for establishing the diagnosis and for guiding potential surgical planning [15].

Characteristic CT findings are not limited to numerical cutoffs but also include distension of the stomach and proximal duodenum, as well as narrowing of the duodenum at the SMA level. Associations with compression of the left renal vein, isolated left renal vein thrombosis, enlargement of the left gonadal vein, or left-sided venous collaterals are additional elements to consider for a positive diagnosis [16]. Rotational CT from the supine to prone position can demonstrate improved outflow past the SMA [17].

Additionally, CT has replaced magnetic resonance enterography (MRE) as the standard investigation [17,18].

The advantage of demonstrating anatomical variants and their consequences, such as delayed transit and obstruction, has been highlighted in limited reports using MRE [19].

Diagnosis can also be confirmed using mesenteric artery ultrasonography [17]. Color Doppler ultrasound can measure the aortomesenteric angle [20]. Endoscopy is useful for diagnosing complications such as esophagitis, reflux gastritis, stasis, and chronic duodenal obstruction [21]. However, suspicion of obstruction of the third part of the duodenum is raised only in some cases during endoscopy [13]. A more specific diagnostic finding is pulsatile extrinsic compression of the duodenum, with exclusion of other diseases of the superior digestive tract [20].

Barium or gastrografin contrast studies are classic diagnostic procedures. Specific findings include dilatation of the stomach and the first and second parts of the duodenum, along with failure of contrast passage through the third part of the duodenum and antiperistaltic flow [13,20]. However, these changes are not specific to SMA syndrome [20].

Gastric-emptying scintigraphy is a well-established imaging modality for evaluating gastroparesis and gastric motility. It may be particularly valuable in differentiating SMA syndrome from gastric motility disorders, especially in patients with diabetes. This investigation provides both qualitative and quantitative assessments of gastric motility, which can aid in distinguishing similar conditions and in evaluating the degree of obstruction or stenosis [20].

Other differential diagnoses include internal hernia, adhesive disease, intussusception [17], and megaduodenum [13].

A challenging situation arises in cases associated with systemic sclerosis, in which gastrointestinal involvement produces a clinical picture similar to SMA syndrome, with progression toward malnutrition [22].

Another rare condition that must be differentiated is aortoduodenal syndrome, defined as obstruction of the third portion of the duodenum by a large abdominal aortic aneurysm [23].

Conservative management is recommended as the initial treatment approach for SMA syndrome [24].

Conservative treatment options include proximal decompression, nutritional support (either enteral or parenteral feeding), correction of metabolic abnormalities, and physical therapy aimed at promoting weight gain and restoring the aortomesenteric fat pad [25].

Total parenteral nutrition or an aggressive enteral nutrition regimen—typically delivered through a nasojejunal tube—may be beneficial. These interventions are designed to increase the volume of the retroperitoneal fat pad, thereby widening the aortomesenteric angle and alleviating symptoms [26]. A psychiatric evaluation is recommended when an eating disorder is suspected [27].

Surgery is considered when conservative measures fail or in patients with a long history of progressive weight loss, marked duodenal dilatation with stasis, or associated complications [28]. Preoperative optimization is essential to correct metabolic imbalances, improve muscle mass, enhance healing potential, and reduce the risk of postoperative complications.

In the reported case, surgical intervention was performed because the patient presented with acute symptoms, duodenal dilatation on CT scan, and a concern for complications; therefore, conservative management could not be applied.

Surgical treatment options include duodenojejunostomy, gastrojejunostomy, and Strong’s procedure (13, 27, 29).

Among these, duodenojejunostomy is the most commonly performed and is regarded as the most effective, providing sustained symptom relief with a low recurrence rate. Gastrojejunostomy is an alternative procedure, though it carries a risk of long-term complications such as bile reflux and ulcer formation [27,29].

Strong’s procedure involves dividing the ligament of Treitz and mobilizing the duodenum to the right of the SMA, thereby avoiding the need for intestinal bypass. It is primarily used in children or in cases where duodenojejunostomy is contraindicated [13,27]. In the reported case, given the patient’s young age, Strong’s procedure was performed.

The optimal timing for transitioning to surgical management remains unclear, as noted by Shin et al. [30].

Nevertheless, surgical intervention may be considered earlier, before the patient’s condition deteriorates or complications arise [13].

Although surgical intervention often results in early symptom relief, long-term follow-up remains essential. Studies of laparoscopic duodenojejunostomy have shown that while most patients experience initial improvement, 21%–66% later develop recurrent symptoms, including dysmotility, gastroparesis, anastomotic stricture, or dumping syndrome [31,32]. Another study reported that 28% of patients ultimately required additional major interventions for persistent motility disorders despite initial improvement, including median arcuate ligament release, colectomy, or even intestinal transplantation [33].

In the reported case, no new complications developed following the surgical intervention, and therefore no additional major procedures were required.

Various complications have been reported in patients with SMA syndrome. The most common complication is gastrointestinal injury, resulting from retained or refluxed gastric and bile acids, as well as elevated intraluminal pressure. The incidence of mucosal injury in SMA syndrome has been reported to range from 25%-59% [34,35].

Unrecognized or severe cases of SMA syndrome may progress to life-threatening complications, including hypovolemic shock, and aspiration pneumonia can also occur. Sudden death has even been reported in young patients. Although the exact mechanisms remain unclear, several hypotheses have been proposed based on published cases and autopsy findings, including arrhythmias due to severe hypokalemia, marked compression of the inferior vena cava by a dilated duodenum, or profound pulmonary depression caused by alkalosis and increased intra-abdominal pressure [34,35]. Therefore, in severe cases, immediate correction of electrolyte and fluid imbalances, along with early reduction of intestinal pressure, is essential.

Inadequately treated or chronic mucosal injuries in SMA syndrome may progress to serious complications such as emphysema, necrosis, portal venous gas, and pneumoperitoneum. Elevated intraluminal pressure in the second portion of the duodenum can disrupt pancreatic juice flow, occasionally resulting in elevated pancreatic enzymes and acute pancreatitis. Since vomiting alone can increase serum amylase—primarily from salivary sources—assessment of pancreatic amylase isoenzymes and lipase is useful for accurately identifying pancreatic involvement.

Recurrent vomiting can also lead to aspiration pneumonia, dehydration, electrolyte imbalances, and severe malnutrition. SMA syndrome may occasionally coexist with other vascular compression disorders [36,37].

Nutcracker syndrome, which is often associated with SMA syndrome, arises from compression of the left renal vein between the SMA and the aorta, leading to impaired renal venous drainage [38,39].

Symptoms of Nutcracker syndrome include hematuria, flank or pelvic pain, anemia, proteinuria, left-sided varicocele in men, and dysmenorrhea in women [38]. Management strategies, depending on the severity of the condition, may include renal vein stenting, renal vein transposition, or autotransplantation [38,39]. In complex cases presenting with significant symptoms, combined duodenal and vascular surgical procedures may be required [39].

Therefore, it is always important to assess for other vascular anomalies in patients diagnosed with SMA syndrome. In the reported case, the patient had no urinary complaints such as hematuria, all renal imaging was normal, and no renal anomalies were observed intraoperatively.

The patient was followed in the ward for 5 days postoperatively and was then discharged with guidance on feeding, warning signs, and scheduled follow-up in the surgical outpatient clinic. At her 2-week and 4-week outpatient visits, she reported no complaints, was feeding well, and had gained weight. She was advised to return for a follow-up appointment after 6 months and was subsequently discharged.

## Conclusion

Despite being a rare cause of obstruction, Wilkie’s syndrome should be considered as a differential diagnosis in patients with chronic illness or a history of prior surgery.

## Patient consent

Informed consent for the publication of this case has been obtained from the patient’s parents.
